# Full-Differential Folded-Cascode Front-End Receiver Amplifier Integrated Circuit for Capacitive Micromachined Ultrasonic Transducers

**DOI:** 10.3390/mi10020088

**Published:** 2019-01-25

**Authors:** Yiheng Du, Changde He, Guowei Hao, Wendong Zhang, Chenyang Xue

**Affiliations:** Key Laboratory of Instrumentation Science & Dynamic Measurement, North University of China, Taiyuan 030051, Shanxi, China; frank_dyheng@163.com (Y.D.); 15536838190@163.com (G.H.); wdzhang@nuc.edu.cn (W.Z.); xuechenyang@nuc.edu.cn (C.X.)

**Keywords:** CMUT, CMOS, operational amplifier, full-differential, transimpedance amplifier, parasitic capacitance, echo-pulse

## Abstract

This paper describes the design of a front-end receiver amplifier for capacitive micromachined ultrasonic transducer (CMUT). The proposed operational amplifier (op amp) consists of a full differential folded-cascode amplifier stage followed by a class AB output stage. A feedback resistor is applied between the input and the output of the op amp to make a transimpedance amplifier. We analyzed the equivalent circuit model of the CMUT element operating in the receiving mode and obtained the static output impedance and center frequency characteristics of the CMUT. The op amp gain, bandwidth, noise, and power consumption trade-offs are discussed in detail. The amplifier was fabricated using GlobalFoundries 0.18-μm complementary metal-oxide-semiconductor (CMOS) technology. The open loop gain of the amplifier is approximately 65 dB, and its gain bandwidth product is approximately 29.5 MHz. The measured input reference noise current was 56 nA/√Hz@3 MHz. The amplifier chip area is 325 μm × 150 μm and the op amp is powered by ±3.3 V, the static power consumption is 11 mW. We verified the correct operation of our amplifier with CMUT and echo-pulse shown that the CMUT center frequency is 3 MHz with 92% fractional bandwidth.

## 1. Introduction

In the field of ultrasonic imaging systems, capacitive micromachined ultrasonic transducers (CMUTs) have attracted a lot of attention owing to their own unique advantages [1,2,3]. Compared with piezoelectric transducers, CMUTs have a higher receiving sensitivity, wider bandwidth, and lower cost, and they are easier to integrate with complementary metal-oxide-semiconductor (CMOS) integrated circuits [4,5,6]. The easy integration characteristics of CMUTs can meet the requirements of developing miniaturized and intelligent imaging systems. In ultrasonic imaging applications, CMUTs can be used for intravascular imaging [7], intracardiac echographies [8], biometric applications [9], underwater imaging [10], and so on. In these ultrasonic imaging applications, obtaining received signal with a high signal-to-noise ratio (SNR) is indispensable for acquiring high resolution images [7,8]. To meet the demands of high SNRs in the field of CMUT-based imaging, specialized receiver circuits should be designed. Optimizing the parameters, such as gain, noise, and bandwidth is the key point to improve the performance and reliability of imaging systems. Many specific amplifier circuits have been introduced in previous studies for the sensed CMUT output current signal [8,9,10,11,12,13,14,15,16]. In [11], a charge amplifier was designed and a strong correlation was verified between simulated and actual CMUT plus circuit performance. To deal with the charge at the floating node, a floating-gate based charge adaptation circuit was used in charge amplifier [12]. In [13], a simple two-stage amplifier topology was designed for 2D CMUT array and the readout amplifier adopted bipolar junction transistors (BJT) input stage and achieved a 75-dB open loop gain. In [14], a low-noise high-gain transimpedance amplifier (TIA) circuit was designed for the CMUT operating between 10 MHz–20 MHz. The TIA circuit adopted a single input stage and a single output stage. A N-metal-oxide-semiconductor (NMOS) transistor was used as the feedback resistance to reduce the parasitic feedback effects, but at the expense of reduced linearity and output swing. The single input stage and single output stage transimpedance circuit topology also have been used in [15,16,17]. Many other different circuit topologies have been proposed, such as [18,19,20], but there was no combined test with CMUT to verify the performance of these topologies. These receiver circuits were primarily designed with gain, bandwidth, and noise specifications and functioned well in their respective cases of application. However, there are still problems such as poor linearity and weak anti-interference ability. In our CMUT front-end receiver circuit system, in order to improve the anti-interference ability and the driving-load ability of the receiver circuit, we decided to employ a fully differential op amp to make a transimpedance amplifier with an external feedback resistor *R*_f_ between the input and the output. To reduce the input reference current noise, we used P-metal-oxide-semiconductor (PMOS) instead of NMOS transistors as the differential input pair. Additionally, rail-to-rail output stage was used to increase the dynamic range of the output voltage and SNR.

In this paper, we present the design of a full-differential folded-cascode operational amplifier for the receiver circuit of a CMUT-based system. The CMUT equivalent circuit model was discussed and the resonant frequency and output static impedance of the CMUT were analyzed. Moreover, the design of our full-differential op amp and noise consideration were presented in detail. Furthermore, the chip layout and our measurement experiments are described and the function of the front-end receiver circuit of CMUT was also verified.

## 2. Capacitive Micromachined Ultrasonic Transducer (CMUT) Element and Equivalent Circuit Model

**Figure 1:**
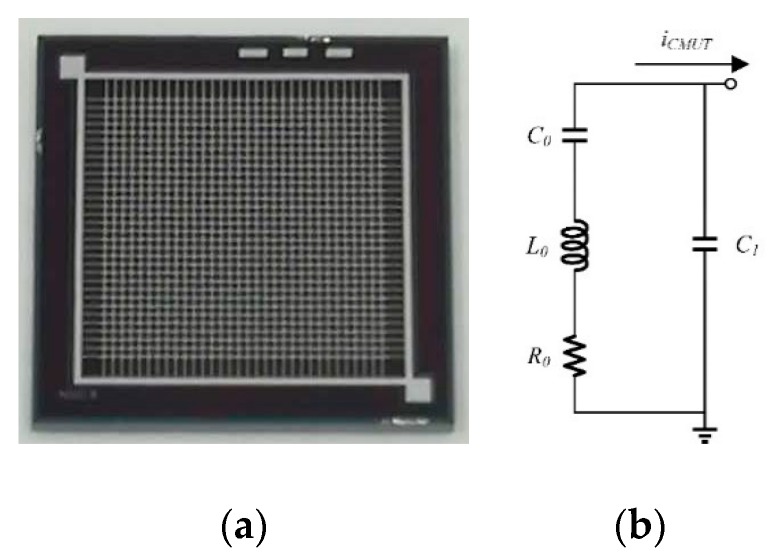
Capacitive micromachined ultrasonic transducer (CMUT) electrical equivalent model. (**a**) A single CMUT element consists of 30 × 30 cells; and (**b**) Butterworth-van-Dyke (BvD) equivalent circuit model of the CMUT element.

The CMUT element is composed of multiple cells connected in parallel, and each cell mainly consists of top and bottom electrodes, membrane, vacuum cavity and insulating layer [10]. The insulation layer can prevent electrical short circuit between the top and bottom electrodes. The CMUT can be used to transmit ultrasonic waves and receive ultrasonic waves, while a direct current (DC) bias voltage is essential. The CMUT is processed by silicon-silicon-on-insulator (Si-SOI) low-temperature bonding technology and a detailed description of this fabrication process can be found in the literature [10]. The membrane size and cavity size of the CMUT with this processing technology are highly controllable and the membrane thickness is consistent. The single CMUT element consists of 30 × 30 cells, and the area of the single CMUT element is 500 μm × 500 μm, as shown in Figure 1a. Since the CMUT is an electrostatically actuated mechanical device, using an electrical equivalent circuit to simulate the mechanical system of the CMUT is an effective method for analyzing its mechanical and electrical properties near the resonant frequency. As shown in Figure 1, we used a four-element Butterworth-van-Dyke (BvD) equivalent circuit to represent the CMUT [18,21,22,23]. The CMUT BvD model has four elements: *C*_0_ is the motional capacitor (inversely proportional to CMUT membrane stiffness), *R*_0_ is the motional resistor (quantifying dissipative losses), *L*_0_ is the motional inductor (proportional to CMUT membrane mass), and *C*_1_ is the electrical capacitance across the CMUT parallel electrodes. The special parameters of the four elements were listed in Table 1.

According to the CMUT BvD equivalent circuit model, the impedance of the CMUT *Z*_CMUT_, is given by:

(1) $$Z_{\mathrm{CMUT}}=\frac{\left(R_{0}+j\omega L_{0}+\frac{1}{j\omega C_{0}}\right)\frac{1}{j\omega C_{1}}}{R_{0}+j\omega L_{0}+\frac{1}{j\omega C_{0}}+\frac{1}{j\omega C_{1}}}$$

The minimum impedance frequency *f*_s_ is given by:

(2) $$f_{s}=\frac{1}{2\pi \sqrt{L_{0}C_{0}}}$$

where *f*_s_ is the series resonant frequency when *L*_0_ resonates with *C*_0_. According to Equations (1) and (2) and Table 1, the output static impedance of the CMUT element is about 5 Ω and resonant frequency of the CMUT is about 5.3 MHz. However, in actual operation, due to the packaging factor of CMUT and the impedance mismatch of sound waves propagating in water, the resonant frequency of actual operation will decrease. It is verified by experiments that the resonant frequency of CMUT under water is about 3 MHz. The output impedance of CMUT in other literatures are dozens kΩ or several MΩ [13,18,21,23], but the output static impedance of our CMUT device is much lower. The low output impedance requires the low input impedance of the receiver circuit to get more sensed current from the CMUT.

## 3. Full-Differential Operational Amplifier Design

**Figure 2:**
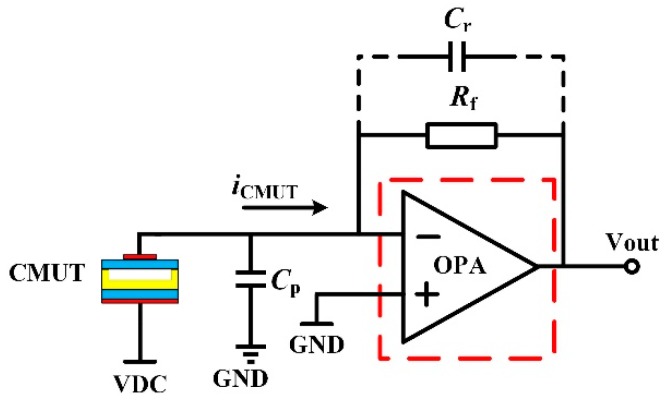
Schematic of the front-end receiver circuit with the CMUT and the red dotted frame is the proposed target amplifier of this design.

When designing the front-end receiver circuit of the CMUT, the impedance of the amplifier should match that of the CMUT to obtain a high SNR. Parameters, including reference input current noise, gain, and bandwidth should accord with the CMUT output current signal. The reference input current noise should be minimized so as to improve the detected sensitivity of current circuit, and the bandwidth of the amplifier and signal speed should be adjusted so as to have a high gain for reducing the influence of subsequent circuit noise on the whole system. A schematic diagram of the front-end receiver circuit with the CMUT is shown in Figure 2. The input CMUT current flows through the feedback resistor *R*_f_ and output voltage signal is generated. The parallel capacitor *C_r_* is used in the circuit to act as low-pass filter (LPF) with *R_f_*.

The transfer function of the circuit shown in Figure 2 is:

(3) $$\frac{V_{out}}{I_{in}}=-\frac{A_{0}}{1+A_{0}}\frac{R_{f}}{1+j\omega C_{p}\frac{R_{f}}{1+A_{0}}}$$

where *C_p_* is the total parasitic capacitance, *R_f_* is the feedback resistance, *A*_0_ is the open loop gain, and A⟶∞, so that the transimpedance gain is approximately equal to *R_f_*.

Additionally:

(4) $$f_{-3dB}=\sqrt{\frac{\mathrm{GBP}}{2\pi R_{f}C_{p}}}$$

where gain bandwidth product (GBP) is of the op amp. For the given GBP and *C_p_*, the feedback resistor *R_f_* determines the values of the output voltage and the −3 dB bandwidth. Moreover, the thermal noise of the feedback resistor also contributes to the input current noise of the receiver circuit, so the value of feedback resistor *R_f_* creates a tradeoff between gain, bandwidth, and noise. Note that the feedback capacitor *C_r_* is not taken into consideration.

**Figure 3:**
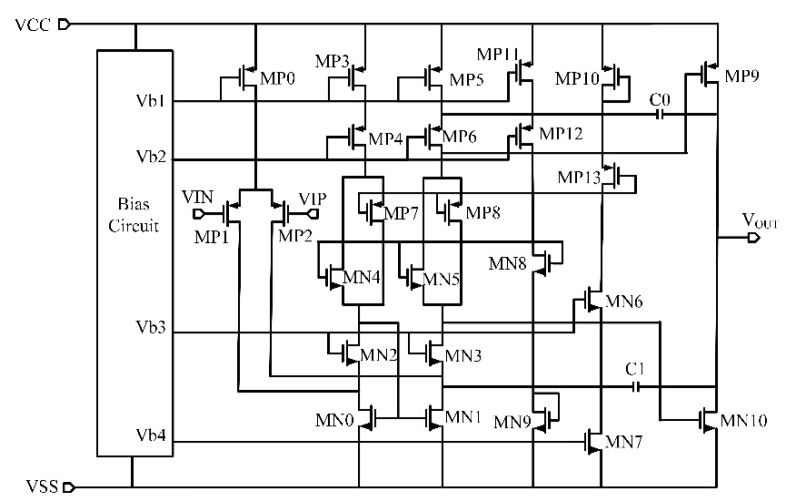
Schematic of the complete two-stage op amp circuit.

We implemented a two-stage full-differential amplifier in the front-end receiver circuit system for CMUT. In order to improve the anti-interference capability of the receiver amplifier circuit, we decided to adopt a fully differential operational amplifier design to make a transimpedance amplifier with a feedback resistor between the input and the output. Compared to [13], to minimize the noise level, the differential input pair uses PMOS instead of NMOS transistors, and a structure with an active load and a folded-cascode stage was adopted to obtain a high gain [24,25,26]. In order to obtain a large output voltage swing and a strong driving ability at the output stage, a rail-to-rail class AB output stage was implemented after the two-stage folded-cascode op amp circuit [27,28,29]. Miller compensation capacitances *C*_0_ and *C*_1_ were adopted between the input and output of amplifier topology to ensure the stability of the amplifier during closed-loop applications and to prevent the occurrence of self-oscillation. The complete two-stage op amp circuit is shown in Figure 3. 

To obtain high detection sensitivity of the CMUT front-end receiver circuit system, a high SNR is indispensable. The input reference current noise of the CMUT and the receiver amplifier circuit are mostly generated by the thermal noise of the feedback resistor *R_f_*, the thermal and flicker noise of the amplifier and thermal noise of the CMUT [15,17,30]. The input reference current noise $\bar{i_{\mathrm{noise}}{}^{2}}$ has been given as:

(5) $$\begin{array}{l} \bar{i_{\mathrm{noise}}{}^{2}}=\frac{\bar{V_{op}{}^{2}}}{{\left(R_{f}//R_{\mathrm{CMUT}}\right)}^{2}}+\bar{i_{op}{}^{2}}+\bar{i_{R_{f}}{}^{2}}+\bar{i_{\mathrm{CMUT}}{}^{2}}\\ =\frac{\bar{V_{op}{}^{2}}}{{\left(R_{f}//R_{\mathrm{CMUT}}\right)}^{2}}+\omega^{2}C_{p}{}^{2}\bar{V_{op}{}^{2}}+\frac{4kT}{R_{f}}+\frac{4kT}{R_{\mathrm{CMUT}}} \end{array}$$

where *R*_CMUT_ is CMUT output impedance and $\bar{V_{op}{}^{2}}$ is the amplifier input reference voltage noise. According to Equation (5), in order to reduce the input current noise, the feedback resistor *R_f_* should be as large as possible and the amplifier input reference voltage noise $\bar{V_{op}{}^{2}}$ should be as low as possible. However, a large feedback resistor would reduce the stability and the dynamic response of the circuit. Thus, the value of *R_f_* signifies a tradeoff between the noise, gain, and stability of the circuit. The amplifier input voltage noise $\bar{V_{\mathrm{op}}{}^{2}}$ is given by [24]:

(6) $$\bar{v^{2}{}_{opa}}=\frac{16kT}{3g_{\mathrm{mP}1}}\left[1+\frac{g_{\mathrm{mbP}1}}{g_{\mathrm{mP}1}}+\frac{1}{g_{\mathrm{mP}1}}\left(g_{\mathrm{mN}0}+g_{\mathrm{mbN}0}\right)+\frac{1}{g_{\mathrm{mP}1}}\left(g_{\mathrm{mP}3}+g_{\mathrm{mbP}3}\right)\right]$$

where *k* is Boltzmann constant, *T* is the temperature, *g*_mP1_, *g*_mN0_ and *g*_mP3_ are the transconductances of MP1, MN0, MP3, respectively, *g*_mbN0_ and *g*_mbP3_ are the substrate transconductances of MN0 and MP3, respectively. Equation (6) represents thermal noise at high frequency of the amplifier and the low frequency 1/f noise of the amplifier is unconsidered here. The second gain noise of the amplifier is also ignored and when referred to the input it is divided by the square of the first stage gain. Since $g_{m}=\sqrt{2\mu C_{ox}\frac{W}{L}I_{D}}$, Equation (6) can be designed with:

(7) $$\bar{v^{2}{}_{opa}}=\frac{16kT}{3\sqrt{2\mu_{p}C_{\mathrm{ox}}{\left(\frac{W}{L}\right)}_{P1}I_{\mathrm{DP}1}}}\left[1+\eta_{P1}+\sqrt{\frac{\mu_{N}{\left(\frac{W}{L}\right)}_{N0}I_{DN0}}{\mu_{p}{\left(\frac{W}{L}\right)}_{P1}I_{\mathrm{DP}1}}}\left(1+\eta_{N0}\right)+\sqrt{\frac{{\left(\frac{W}{L}\right)}_{P3}I_{\mathrm{DP}3}}{{\left(\frac{W}{L}\right)}_{P1}I_{\mathrm{DP}1}}}\left(1+\eta_{P3}\right)\right]$$

where $\eta_{P1}=\frac{g_{\mathrm{mbP}1}}{g_{\mathrm{mP}1}}$, $\eta_{N0}=\frac{g_{\mathrm{mbN}0}}{g_{\mathrm{mN}0}}$, $\eta_{P3}=\frac{g_{\mathrm{mbP}3}}{g_{\mathrm{mP}3}}$, *I*_D_ is drain source current. Since *I*_DN0_ = 2*I*_DP1_ = 2*I*_DP3_, so, to decrease the amplifier input voltage noise, ${\left(\frac{W}{L}\right)}_{P1}$ and $I_{\mathrm{DP}1}$ should be increased, make $2\mu_{N}{\left(\frac{W}{L}\right)}_{N0}$ < $\mu_{p}{\left(\frac{W}{L}\right)}_{P1}$ and make ${\left(\frac{W}{L}\right)}_{P3}$ < ${\left(\frac{W}{L}\right)}_{P1}$.The large $I_{\mathrm{DP}1}$ increase the power consumption of the circuit and large ${\left(\frac{W}{L}\right)}_{P1}$ will increase the gain of the circuit and reduce the noise while reducing the bandwidth. Thus, these parameters are compromised during the circuit design process. 

## 4. Experimental Results and Discussion

**Figure 4:**
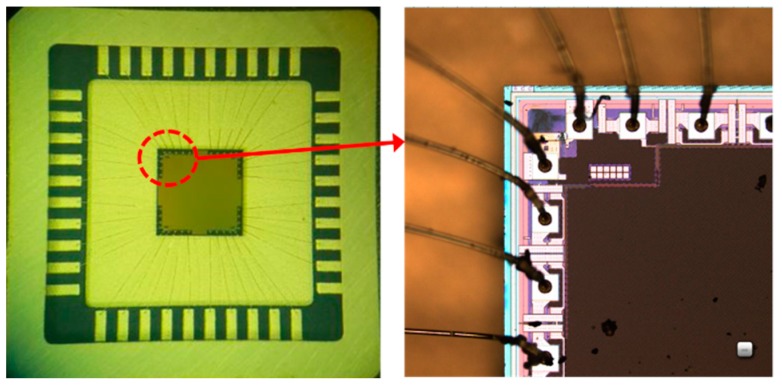
Amplifier chip in low-profile quad flat package (LQFP) package.

The chip layout was done using GlobalFoundries 0.18-μm CMOS technology. The size of op amp chip is 325 μm × 150 μm, as shown in Figure 4. In order to improve the stability and reliability of the circuit chip, passivation layer is added on the chip surface to protect the internal circuit structure and prevent circuit damage. For the convenience and reliability of the chip testing, the chip is packged by low-profile quad flat package (LQFP) standard packaging technology.

**Figure 5:**
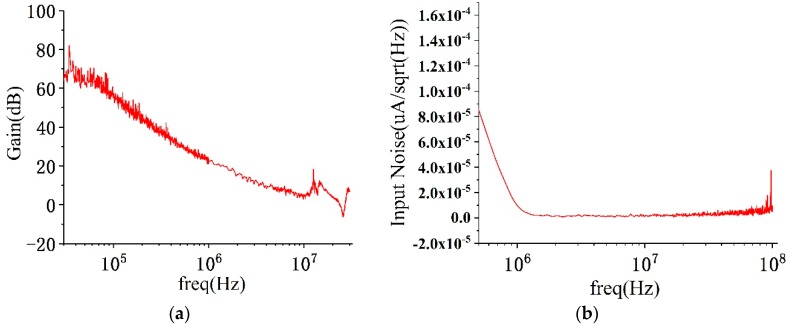
Measurements of the amplifer chip performance. (**a**) The result of open-loop gain; and (**b**) measurement of the input reference noise current.

We tested the open loop gain of the amplifier chip using an Agilent Network Analyzer E5061B (Agilent Technologies Inc, Santa Clara, CA, USA). In order to prevent damage to the instrument caused by excessive power, we used an attenuation probe during testing. The measured open loop gain was approximately 65 dB, and the GBP was approximately 29.5 MHz as shown in Figure 5a. The gain trace fluctuations in the high-gain region were caused by a decrease in dynamic performance due to the 20 dB loss of the attenuation probe. The output reference current noise was measured using an Agilent spectrum analyzer N9030A (Agilent Technologies Inc.), and the measured output current noise was divided by the transfer function to obtain input reference current noise. The measured output reference current noise was 11.87 nA/√Hz@3 MHz, and the input reference current noise was 56 nA/√Hz@3 MHz, as shown in Figure 5b. The chip was powered by ±3.3 V, and the static power consumption was 11 mW. 

**Figure 6:**
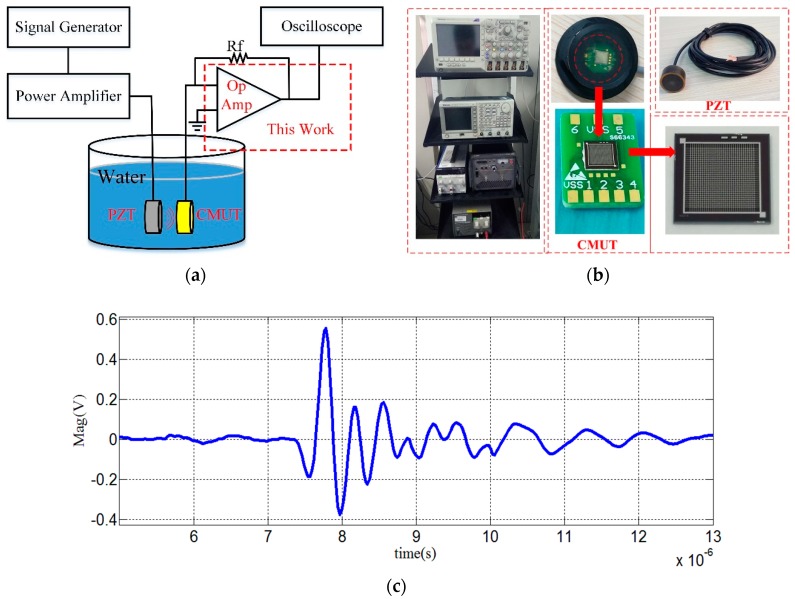
Functional test of the amplifier with the encapsulated CMUT. (**a**) Schematic diagram of the CMUT receiver circuit for the functional test; (**b**) measurement setup for the amplifier with an encapsulated CMUT and a PZT; and (**c**) the received signal of the CMUT.

To test the function of the proposed amplifier circuit to convert the output current of the CMUT to voltage, the CMUT was operated in the receiving mode, as shown in Figure 6a. Assuming that the total parasitic capacitance of printed circuit board (PCB) and wire is 10 pF, the input capacitance of the CMUT was approximately 905 pF, which limited the bandwidth of the front-end receiver circuit in terms of parasitic capacitance. So, the total parasitic capacitance is about 915 pF. In order to meet the CMUT bandwidth requirement (2–5 MHz) based on Equation (4), the feedback resistor *R_f_* was chosen to be 200 Ω with a 5 MHz bandwidth. We used a standard piezoelectric transducer (PZT) to transmit ultrasonic signals and the encapsulated CMUT device to receive the signals. A 30-V DC bias voltage was applied at the bottom electrode of the CMUT. The received signal is shown in Figure 6c. It should be noted that since the static capacitance of the CMUT itself limited the bandwidth in the form of parasitic capacitance, the feedback resistor cannot be very large. Compared to [11] with full differential charge amplification, this design used fully differential transimpedance amplification to achieve 46 dB transimpedance gain, much higher than [11], but lower than the single input transimpedance amplifier [13,14,15]. However, this problem could be solved if we consider to increase the GBP of the amplifier to get higher transimpedance gain under bandwidth constraints. The higher impedance gain also will increase the SNR of the receiver system. Moreover, in [13,14,15], when the input current changes, the static operating point of the circuit would change accordingly, resulting in heavily nonlinear whenever the input current rises to high value. But in this paper, the op amp used class AB rail-to-rail output stage, the static operating point is constant and will not change with the input signal. Thus, the propose op amp has a better linearity than [13,14,15]. Additionally, the full-differential op amp has a better anti-interference ability than single-end amplifier [24].

**Figure 7:**
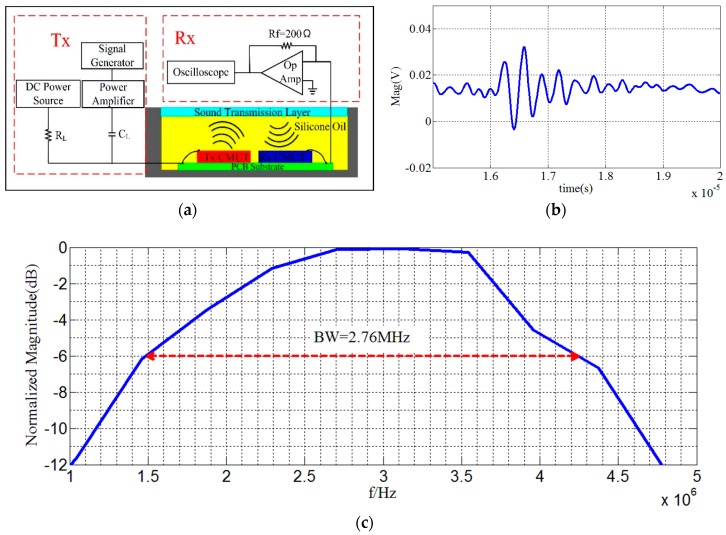
Transceiver testing of CMUT. (**a**) Schematic diagram of transceiver testing circuit; (**b**) echo signal of the CMUT; and (**c**) the normalized frequency response of the pulse-echo.

The working mechanism of the integrated circuit test system of the transceiver is illustrated in Figure 7a. Both of high frequency single pulsed alternating current (AC) voltage and 30-V DC bias voltage were applied in the bottom electrode of Tx CMUT. The Rx CMUT sensed pulse-echo signal and current signal via the top electrode. Figure 7b,c show the echo signal and the normalized frequency response of the pulse-echo, respectively. Here we can see, the −6 dB center frequency is 3 MHz and the transducer has a bandwidth of 2.76 MHz with a relative bandwidth of 92%.

## 5. Conclusions

We have presented the design and verification of a full-differential folded-cascode operational amplifier for CMUT front-end receiver circuit. In the operational amplifier design, the trade-offs between the gain, bandwidth, noise performance, and the power consumption are discussed in detail. By analyzing the BvD equivalent circuit model of the CMUT, the static output impedance and operating frequency of the CMUT element were obtained to match the front-end receiver circuit. Since the static capacitance of the CMUT itself limited the bandwidth in the form of parasitic capacitance, the receiver circuit was designed to meet the bandwidth constrain at the expense of the transimpedance gain. Methods are available to improve the transimpedance gain for future designs. We verified the correct operation of the amplifier via experiments involving receiver and transceiver tests with the CMUT device. It was shown that the CMUT center frequency is 3 MHz with 92% fractional bandwidth.

## Figures and Tables

**Table 1:** Parameters of the capacitive micromachined ultrasonic transducer (CMUT) element electrical equivalent model.

Component	Value
Motional capacitor *C*_0_ (pF)	831.88
Motional inductor *L*_0_ (μH)	1.02
Motional resistor *R*_0_ (Ω)	5.30
Electrical capacitance *C*_1_ (pF)	73.30

## References

[1] Oralkan O., Ergun A.S., Johnson J.A., Karaman M., Demirci U., Kaviani K., Lee T.H., Khuri-Yakub B.T. (2002). Capacitive micromachined ultrasonic transducers: Next-generation arrays for acoustic imaging?. IEEE Trans. Ultrason. Ferroelectr. Freq. Control.

[2] Khuri-Yakub B.T., Oralkan O. (2011). Capacitive micromachined ultrasonic transducers for medical imaging and therapy. J. Micromech. Microeng..

[3] Salim M.S., Abd Malek M.F., Heng R.B.W., Juni K.M., Sabri N. (2012). Capacitive micromachined ultrasonic transducers: Technology and application. J. Med. Ultrason..

[4] Ergun A.S., Yaralioglu G.G., Khuri-Yakub B.T. (2003). Capacitive micromachined ultrasonic transducers: Theory and technology. J. Aerosp. Eng..

[5] Jeong B.G., Kim D.K., Hong S.W. (2013). Performance and reliability of new CMUT design with improved efficiency. Sens. Actuat. A-Phys..

[6] Yongli H., Ergun A.S., Haggstrom E., Badi M.H., Khuri-Yakub B.T. (2003). Fabricating capacitive micromachined ultrasonic transducers with wafer-bonding technology. J. Microelectromech. Syst..

[7] Yeh D.T., Oralkan O., Wygant I.O., O’Donnell M., Khuri-Yakub B.T. (2006). 3-D ultrasound imaging using a forward-looking CMUT ring array for intravascular/intracardiac applications. IEEE Trans. Ultrason. Ferroelectr..

[8] Gurun G., Tekes C., Zahorian J., Xu T., Satir S., Karaman M., Hasler J., Degertekin F.L. (2014). Single-chip CMUT-on-CMOS front-end system for real-time volumetric IVUS and ICE imaging. IEEE Trans. Ultrason. Ferroelectr..

[9] Iula A., Savoia A., Caliano G. (2011). Capacitive micro-fabricated ultrasonic transducers for biometric applications. Microelectron. Eng..

[10] Song J.L., Xue C.Y., He C.D., Zhang R., Mu L.F., Cui J., Miao J., Liu Y., Zhang W.D. (2015). Capacitive micromachined ultrasonic transducers (CMUTs) for underwater imaging applications. Sensors.

[11] Noble R.A., Davies R.R., King D.O., Day M.M., Jones A.R.D., Mcintosh J.S., Hutchins D.A., Saul P. Low-temperature micromachined cMUTs with fully-integrated analogue front-end electronics. Proceedings of the IEEE Ultrasoncs Symposium.

[12] Peng S.Y., Qureshi M.S., Basu A., Guldiken R.O., Degertekin F.L., Hasler P.E. Floating-gate based CMUT sensing circuit using capacitive feedback charge amplifier. Proceedings of the IEEE Ultrasoncs Symposium.

[13] Cicek I., Bozkurt A., Karaman M. (2005). Design of a front-end integrated circuit for 3D acoustic imaging using 2D CMUT arrays. IEEE Trans. Ultrason. Ferroelectr..

[14] Wygant I.O., Zhuang X., Yeh D.T., Oralkan O., Sanli Ergun A., Karaman M., Khuri-Yakub B.T. (2008). Integration of 2D CMUT arrays with front-end electronics for volumetric ultrasound imaging. IEEE Trans. Ultrason. Ferroelectr. Freq. Control.

[15] Gurun G., Hasler P., Degertekin F. (2011). Front-end receiver electronics for high-frequency monolithic CMUT-on-CMOS imaging arrays. IEEE Trans. Ultrason. Ferroelectr. Freq. Control.

[16] Nikoozadeh A., Wygant I.O., Lin D.S., Oralkan O., Ergun A.S., Stephens D.N., Thomenius K.E., Dentinger A.M., Wildes D., Akopyan G. (2008). Forward-looking intracardiac ultrasoundimaging using a 1-D CMUT array integrated with custom front-end electronics. IEEE Trans. Ultrason. Ferroelectr..

[17] Huang X., Cheong J.H., Cha H.K., Yu H., Je M., Yu H. A high-frequency transimpedance amplifier for CMOS integrated 2D CMUT array towards 3D ultrasound imaging. Proceedings of the 35th Annual International Conference of the IEEE Engineering in Medicine and Biology Society (EMBC).

[18] Sharma S., Ytterdal T. Low noise front-end amplifier design for medical ultrasound imaging applications. Proceedings of the IEEE-IFIP International Conference on VLSI and System-on-Chip.

[19] Behnamfar P., Molavi R., Mirabbasi S. (2016). Transceiver design for CMUT-based super-resolution ultrasound imaging. IEEE Trans. Biomed. Circ. Syst..

[20] Cenkeramaddi L.R. Feedback biasing based adjustable gain ultrasound preamplifier for CMUTs in 45 nm CMOS. Proceedings of the International Conference on Vlsi Design and 2018 International Conference on Embedded Systems.

[21] Kumar M., Seok C., Mahmud M.M., Zhang X., Oralkan O. A low-power integrated circuit for interfacing a capacitive micromachined ultrasonic transducer (CMUT) based resonant gas sensor. Proceedings of the 2015 IEEE SENSORS.

[22] Lohfink A., Eccardt P.C. (2005). Linear and nonlinear equivalent circuit modeling of CMUTs. IEEE Trans. Ultrason. Ferroelectr. Freq. Control.

[23] Cenkeramaddi L.R., Bozkurt A., Yamaner F.Y., Ytterdal T. A low noise capacitive feedback analog front-end for CMUTs in Intra Vascular ultrasound imaging. Proceedings of the Ultrasonics Symposium.

[24] Razavi B. (2001). Design of Analog CMOS Integrated Circuits.

[25] Mallya S., Nevin J.H. (1989). Design procedures for a fully differential folded-cascode CMOS operational amplifier. IEEE J. Solid-State Circuits.

[26] Daoud H., Salem S.B., Zouari S., Loulou M. Folded cascode OTA design for wide band applications. Proceedings of the International Conference on Design and Test of Integrated Systems in Nanoscale Technology.

[27] Bogner P., Habibovic H., Hartig T. A high signal swing Class AB earpiece amplifier in 65nm CMOS Technology. Proceedings of the 32nd European Solid-State Circuits Conference.

[28] Rincon-Mora G.A., Stair R. (2001). A low voltage, rail-to-rail, class AB CMOS amplifier with high drive and low output impedance characteristics. IEEE Trans. Circuits Syst. II Analog Dig. Signal Process..

[29] Lu C.W. (2009). A Rail-To-Rail Class-AB Amplifier with an Offset Cancellation for LCD Drivers. IEEE J. Solid-State Circuits.

[30] Cheng T.C., Tsai T.H. (2016). CMOS Ultrasonic Receiver with On-Chip Analog-to-Digital Front End for High-Resolution Ultrasound Imaging Systems. IEEE Sens. J..

